# Gap Junction Mediated Intercellular Metabolite Transfer in the Cochlea Is Compromised in Connexin30 Null Mice

**DOI:** 10.1371/journal.pone.0004088

**Published:** 2008-12-31

**Authors:** Qing Chang, Wenxue Tang, Shoeb Ahmad, Binfei Zhou, Xi Lin

**Affiliations:** 1 Department of Otolaryngology, Emory University School of Medicine, Atlanta, Georgia, United States of America; 2 Department of Cell Biology, Emory University School of Medicine, Atlanta, Georgia, United States of America; National Institutes of Health, United States of America

## Abstract

Connexin26 (Cx26) and connexin30 (Cx30) are two major protein subunits that co-assemble to form gap junctions (GJs) in the cochlea. Mutations in either one of them are the major cause of non-syndromic prelingual deafness in humans. Because the mechanisms of cochlear pathogenesis caused by Cx mutations are unclear, we investigated effects of Cx30 null mutation on GJ-mediated ionic and metabolic coupling in the cochlea of mice. A novel flattened cochlear preparation was used to directly assess intercellular coupling in the sensory epithelium of the cochlea. Double-electrode patch clamp recordings revealed that the absence of Cx30 did not significantly change GJ conductance among the cochlear supporting cells. The preserved electrical coupling is consistent with immunolabeling data showing extensive Cx26 GJs in the cochlea of the mutant mice. In contrast, dye diffusion assays showed that the rate and extent of intercellular transfer of multiple fluorescent dyes (including a non-metabolizable D-glucose analogue, 2-NBDG) among cochlear supporting cells were severely reduced in Cx30 null mice. Since the sensory epithelium in the cochlea is an avascular organ, GJ-facilitated intercellular transfer of nutrient and signaling molecules may play essential roles in cellular homeostasis. To test this possibility, NBDG was used as a tracer to study the contribution of GJs in transporting glucose into the cochlear sensory epithelium when delivered systemically. NBDG uptake in cochlear supporting cells was significantly reduced in Cx30 null mice. The decrease was also observed with GJ blockers or glucose competition, supporting the specificity of our tests. These data indicate that GJs facilitate efficient uptake of glucose in the supporting cells. This study provides the first direct experimental evidence showing that the transfer of metabolically-important molecules in cochlear supporting cells is dependent on the normal function of GJs, thereby suggesting a novel pathogenesis process in the cochlea for Cx-mutation-linked deafness.

## Introduction

Gap junctions (GJs) are the only known intercellular channels linking the cytoplasm of adjacent cells. They facilitate intercellular exchanges of ion, metabolite and signaling molecules. GJs in all vertebrates are assembled from connexins (Cxs). Six compatible Cx subunits assemble to form a GJ hemichannel in the cell membrane, and two GJ hemichannels align with their extracellular domains to form a complete GJ intercellular channel. The Cx gene family has twenty-one and twenty members in human and mouse genomes, respectively [1]. Genetic studies have linked many human diseases to Cx mutations [2]. Mutations in Cx26 [3], [4], [5] and Cx30 [6] genes are the most common genetic defects found in patients suffering inherited nonsyndromic deafness. The essential role that Cxs play in hearing is further recognized by profound deafness displayed in Cx30 null and conditional Cx26 null mouse models [7], [8]. It is clear that normal functions of Cx26 and Cx30 are required for hearing. However, the molecular and cellular mechanisms explaining why these two Cxs are essential for normal hearing are unclear, although several plausible theories have been proposed [9], [10].

GJ-mediated ionic transfer is termed *ionic coupling*, while exchanges of larger molecules that are usually not permeable through traditional ion channels are called *metabolic* or *biochemical coupling* [11]. GJ-mediated ionic diffusion has been considered an essential mechanism to allow endolymphatic K^+^ to recycle in the cochlea in order to maintain the high extracellular K^+^ concentration (∼160 mM) in the endolymph. Both the high K^+^ concentration and the accompanying electrical potential (endolymphatic potential) in the endolymphatic space are required for normal hearing [12]. Disruption of such a constant ionic flow in the cochlea by loss-of-function Cx mutations has been a leading theory in the field of auditory physiology explaining deafness caused by Cx mutations [9]. However, direct experimental evidence in supporting of such a hypothesis is not available.

Recent data reveal that most cochlear GJs are co-assembled from Cx26 and Cx30 [13], [14]. Therefore, deleting a single Cx gene is unlikely to totally eliminate intercellular ionic coupling since the remaining Cx gene is still able to function and form homomeric GJs. This notion is directly supported by data showing that the cellular expression patterns of Cx30 and Cx26 in the cochlea of Cx26 and Cx30 null mice, respectively, are unaltered [7], [8]. Furthermore, *in vitro* studies of reconstituted GJs reveal that a subset of Cx26 point mutations specifically affecting biochemical coupling (e.g., V84L, V95M and A88S) are sufficient to cause deafness in human patients [15], [16]. These recent findings suggest that substantial ionic coupling remains in the cochlea of Cx30 null mice, which are inconsistent with the K^+^ recycling theory. Therefore, further investigations into cellular mechanisms of cochlear pathogenesis resulted from Cx mutations are needed.

**Figure 1:**
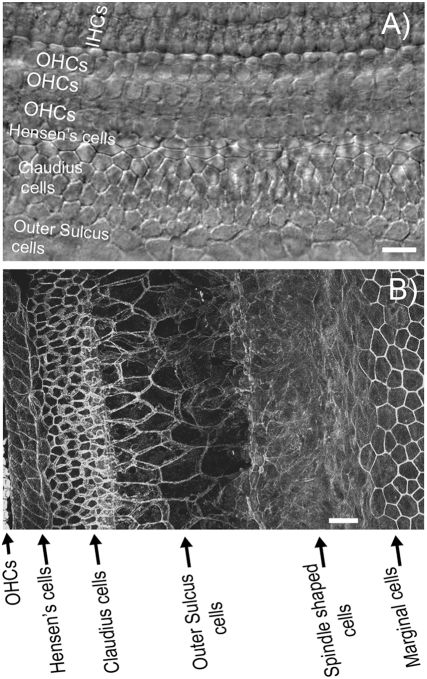
Illustration of the flattened cochlear preparation used in this study. (A) DIC image of an unfixed live preparation. Various types of cochlear cells are labeled. These cells are identified by their distinct shape and relative location in the cochlea. (B) A flattened cochlear preparation fixed and stained with phalloidin to outline the cell borders. Cochlear supporting cells, cells in the lateral wall and stria vascularis are labeled according to their distinct shape and relative location. Scale bars represent approximately 100 µm. Abbreviations: IHCs, inner hair cells; OHC, outer hair cells.

Similar to the lens in the eye, where mutations in Cx46 and Cx50 cause cataracts [11], [17], the sensory epithelium in the cochlea is an avascular organ. While the extensive intercellular coupling provided by GJs between cochlear supporting cells is thought to play nutritive roles in maintaining cellular homeostasis [18], it has yet to be thoroughly investigated. Therefore we used a novel flattened cochlear preparation (Fig. 1) to directly assess GJ-mediated ionic and metabolic coupling in the sensory epithelium of the cochlea. By comparing wild type (WT) and Cx30 null mice, our results provide direct experimental evidence demonstrating that targeted deletion of Cx30 significantly compromised intercellular transfer of metabolically-important molecules among the supporting cells in the cochlea. In contrast, GJ conductance measured by two-electrode patch clamp recordings among these cells was not significantly different. The large reduction in the GJ-mediated transfer of a D-glucose analogue suggested that cellular energy supply was diminished in the cochlear sensory epithelium that normally lacks adequate microcirculation. Our data have identified one important cellular deficit that potentially constitutes a major pathogenesis process, namely a reduction in GJ-mediated glucose transfer in the cochlea of Cx30 mutant mice. These data support a novel theory explaining Cx-mutation-linked hearing loss.

## Results

**Figure 2:**
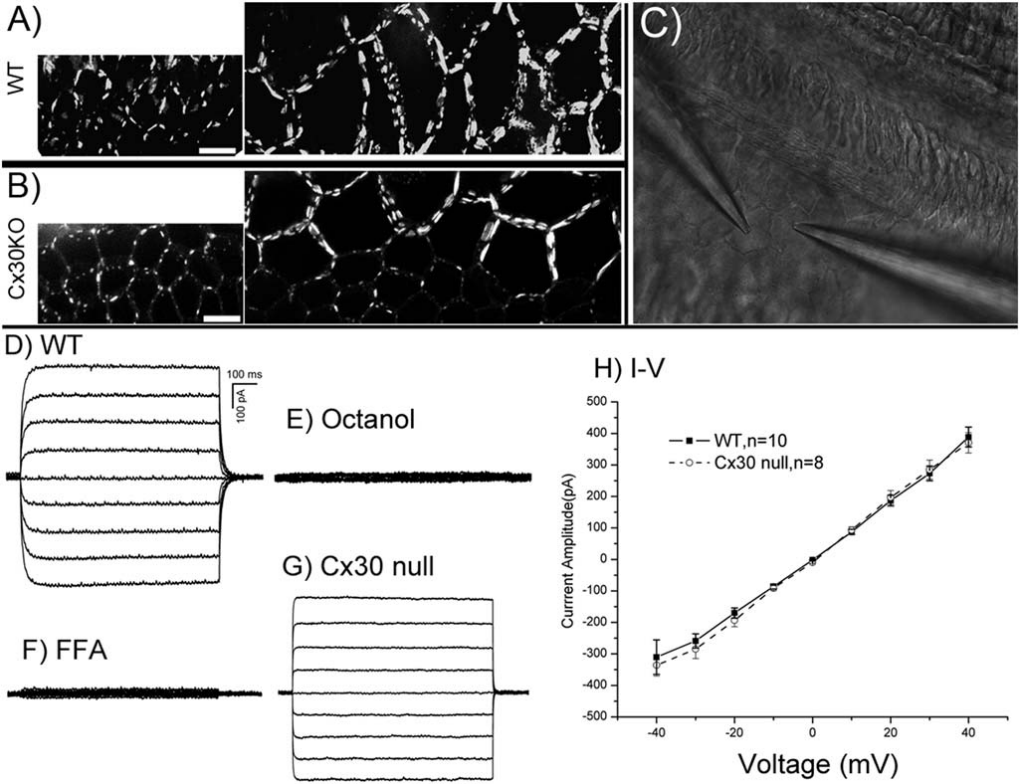
Assessment of ionic coupling in the supporting cells of WT and Cx30 null mice. (A) Patterns of Cx26 immunoreactivities in the Claudius (left panel) and outer sulcus (right panel) cells of WT mice. (B) Patterns of Cx26 immunoreactivities in the Claudius (left panel) and outer sulcus (right panel) cells of Cx30 null mice. Scale bars represent approximately 100 µm. (C) An image showing the setup of two-electrode patch clamp recording performed with the flattened cochlear preparation. A pair of outer sulcus cells was recorded in this example. (D) Transjunctional whole cell currents recorded from WT mice. Scale bars apply to figures in D–G. (E) Transjunctional whole cell currents recorded from WT mice in the presence of 2 mM octanol. (F) Transjunctional whole cell currents recorded from WT mice in the presence of 200 µM FFA. (G) Transjunctional whole cell currents recorded from Cx30 null mice. (H) The peak amplitude of whole cell transjunctional currents is plotted as a function of transjunctional voltage (V_j_) to obtain the I–V relations for WT (solid line) and Cx30 null (dashed line) mice.

The sensory epithelium of the cochlea does not contain microvessels [19], yet the density of GJ plaques in cochlear supporting cells is exceptionally high [14], [20]. The intercellular coupling provided by these GJs is suspected to play nutritive roles by facilitating local glucose and other metabolite transfers [18]. We, therefore, concentrated our effort on comparing differences in GJ-mediated ionic and metabolic coupling in the cochlear supporting cells between the WT and Cx30 null mice. We first examined whether the Cx30 null mutation affects the formation of GJs in this region of the cochlea by immunolabeling for Cx26 and Cx30 and examining the results using the flattened cochlear preparation. Consistent with the genetic deletion of the *Gjb6* (Cx30) gene, no Cx30 immunoreactivities were detected (Fig. S1B). Previous studies using Western blotting also confirmed that these mice do not express the Cx30 protein [8], [21]. Extensive Cx26 immunoreactivities clustered apparently as GJ plaques, however, were found in the membrane of cochlear supporting cells of Cx30 null mice (Fig. 2B & Fig. S1A). Comparing the size of the evident GJ plaques between WT (Fig. 2A) and Cx30 null (Fig. 2B) mice, the density of Cx26 immunoreactivities in the Cx30 null mice was apparently decreased. Nonetheless, these presumably homomeric Cx26 GJ plaques were observed in the membrane of all cochlear supporting cells that normally co-express Cx26 and Cx30 (Fig. 2B). These results were consistent with previously published data showing that the cellular expression pattern of Cx26 is unaltered in the cochlear sections of Cx30 null mice [7], [8]. Next, we quantified the effect of the Cx30 null mutation on intercellular ionic coupling by measuring GJ conductance between adjacent cochlear supporting cells using two-electrode patch clamp recordings conducted with the flattened cochlear preparation (Fig. 2C). The intercellular currents recorded in WT mice (Fig. 2D) were eliminated by GJ blockers (2 mM of octanol (Fig. 2E) or 200 µM flufenamic acid (FFA, Fig. 2F)), indicating that GJs mediated the electrical coupling. A significant amount of GJ-mediated current remained in the Cx30 null mice (Fig. 2G). Average GJ conductance (G_j_) derived from the I–V relation was 9.1±2.2 nS (the total number of cells (n) recorded was 10) and 8.3±1.3 nS (n = 8) in WT and Cx30 null mice, respectively (Fig. 2H). The small reduction of GJ conductance in the Cx30 null mice was statistically not significant (p>0.05). These whole cell recording data indicate that the absence of Cx30 did not appreciably reduce ionic coupling among cochlear supporting cells.

**Figure 3:**
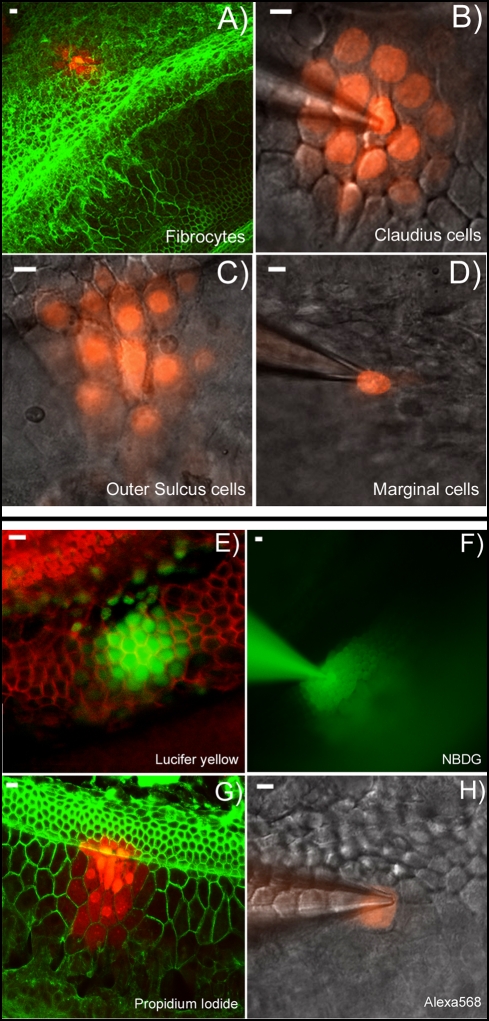
Validation of the dye diffusion assay performed with the flattened cochlear preparation. A–D: Dye diffusion patterns after PI was injected into a single cell in various locations in the cochlea. The type of the cells that was injected is given at lower right corner of each panel. E–F: Diffusion patterns of four different fluorescent dyes after injecting into a single Claudius cell. Name of the dye is given in the lower right corner of each panel. Panels B), C), D), F) & H) were photographed with unfixed fresh samples. Panels A), E), G) were results obtained from fixed samples after the experiments were done. They were labeled with fluorescent phalloidin (red in E, green in A&G) to outline the cell border. Scale bar on the top left of each panel represents approximately 100 µm.

We next investigated whether the absence of Cx30 affected GJ-mediated metabolic coupling among cochlear supporting cells by using dye diffusion assays performed with the flattened cochlear preparation. This approach was first validated by performing dye injections with propidium iodide (PI) into single cells at various locations in the cochlea of WT mice. PI is a well-characterized fluorescent dye known to pass through cochlear GJs [15]. The dye was readily transferred to multiple cells when single cell injections were made into fibrocytes located in the lateral wall (Fig. 3A, n = 10), Claudius cells (Fig. 3B, n = 18) and outer sulcus cells (Fig. 3C, n = 26). As a negative control, we injected PI into the marginal cells since these cells are not coupled by GJs [13], [14]. For all the marginal cells we tested (n = 8), the dye stayed in the injected cell for more than 30 minutes (Fig. 3D). Two other fluorescent dyes (e.g., Lucifer yellow (LY), 2-NBDG) used in this study showed similar results when injected into marginal cells. The failure of intercellular transfer of the membrane impermeable dye among marginal cells supports the hypothesis that GJs mediate the intercellular diffusion of these fluorescent dyes.

**Figure 4:**
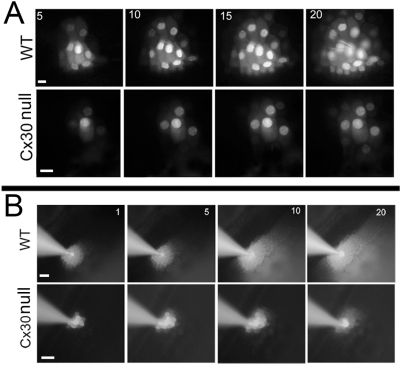
Time-lapse recordings of intercellular dye transfer in WT and Cx30 null mice after fluorescent dye was injected into a single outer sulcus cell. (A) Comparison of intercellular PI diffusion patterns in WT (upper row panels) and Cx30 null (lower row panels) mice. Time (in minutes) after the dye was injected into a single cell is given by the numbers in the top left corner of pictures. (B) Comparison of intercellular 2-NBDG diffusion patterns in WT (upper row panels) and Cx30 null (lower row panels) mice. Time (in minutes) after the dye injection is given in the top right corner of the pictures. Scale bars represent approximately 100 µm.

We extended the *in situ* dye injection assay by injecting fluorescent dyes possessing different charges and molecular weights. In Claudius and outer sulcus cells of WT mice, both negatively (LY, Fig. 3E) and positively charged (PI, Fig. 3G) dyes diffused to many cells in a few minutes. Alexa 568 (MW = 791.8, Charge = −1), in contrast, showed minimal intercellular diffusion among the WT cochlear supporting cells (Fig. 3H, n = 10). This dye apparently represented an upper limit for a negative-charged dye to be transferred across cochlear GJs. Alternatively, a fluorescent dye commonly used to evaluate intercellular glucose transfer, 2-NBDG [22], showed remarkably fast diffusion among cochlear supporting cells. Both the rate and extent of diffusion of 2-NBDG were dramatically greater than those demonstrated by LY and PI (Fig. 3F, Fig. 4).

**Figure 5:**
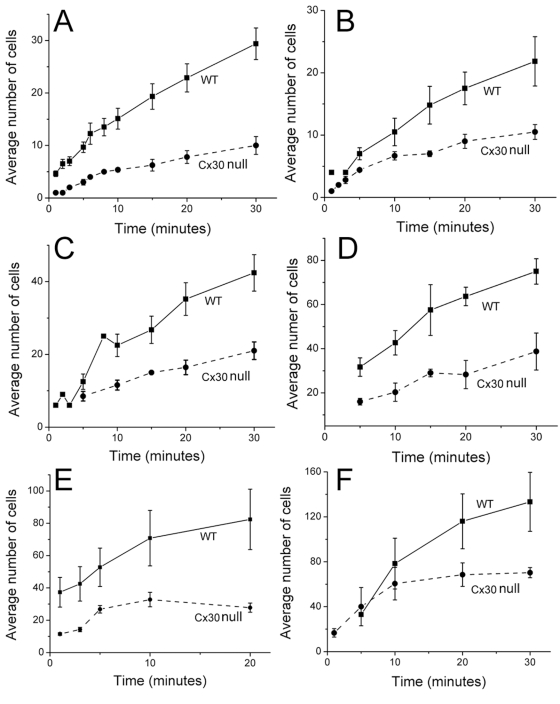
Comparison of the number of cells (y-axis) received fluorescent dye through GJ-mediated diffusion in WT and Cx30 null mice. X-axis gives the time after the establishment of whole cell recording configuration. Three fluorescent dyes, PI (A&B), LY (C&D), and 2-NBDG (E&F) were tested in the study. Panels in the left column (A, C&E) summarize the data obtained from injections into a single Claudius cell. Panels in the right column (B, D&F) summarize the data obtained from injections into a single outer sulcus cell. Solid lines are data obtained from WT animals. Dashed lines are data obtained from Cx30 null mice. Vertical bars represent standard error of the mean.

After validating the *in situ* dye injection assay, we compared the efficiency of intercellular biochemical coupling among cochlear supporting cells of WT and Cx30 null mice. To quantify differences in metabolic coupling among these cells, we performed time-lapsed recordings to assess the time course of PI (Fig. 4A) and 2-NBDG (Fig. 4B) diffusion. Data obtained from WT and Cx30 null mice at various time points after the start of dye injections showed that the extent of the fluorescent dye diffusion gradually increased with time (Fig. 4 A&B). Quantifying these data by plotting the number of dye recipient cells as a function of time after the injection indicates that for both the positive (PI, Fig. 5 A&B) and negative (LY, Fig. 5 C&D) dyes, the number of cells receiving dye transfer in the sensory epithelium of Cx30 null mice was consistently less at all time points. 2-NBDG showed an exceptional ability to pass through cochlear GJs among the supporting cells. Injections of 2-NBDG into a single Claudius cell quickly diffused to nearly 40 cells within 1 minute (Fig. 5E). On average, about 80 cells received the D-glucose analogue through the GJ-mediated diffusion in about 20 minutes in the WT mice. In contrast, less than 30 cells received 2-NBDG in the cochlea of Cx30 null mice during the same time period (Fig. 5E). Similar differences were observed between the WT and Cx30 null mice when 2-NBDG was injected into a single outer sulcus cell (Fig. 5F). These data demonstrated that Cx30 null mutation caused a deficit in GJ-mediated metabolite transfer among cells in the sensory epithelium of the cochlea.

**Figure 6:**
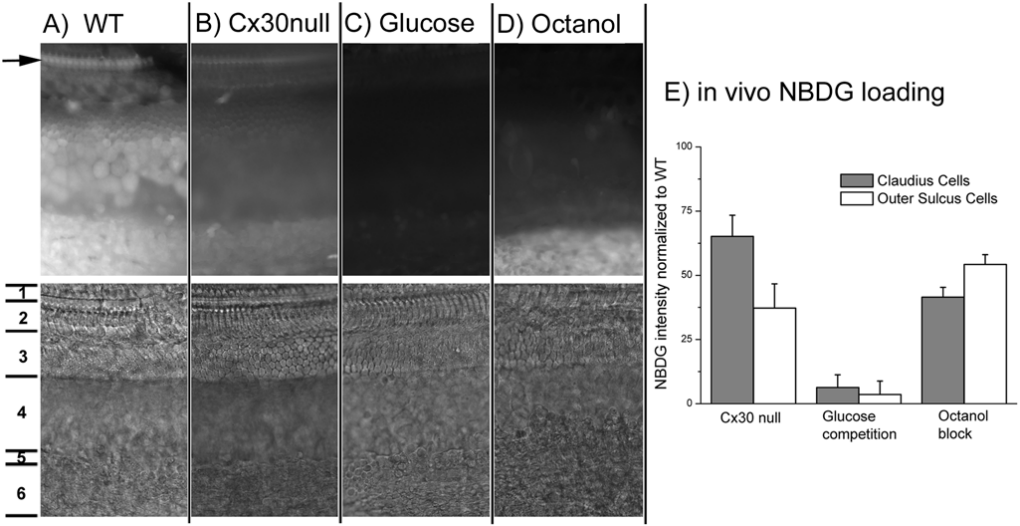
Use of 2-NBDG as a fluorescent tracer of glucose to study the dependence of glucose uptake by cochlear supporting cells on the GJs. A)–D) Fluorescent images of 2-NBDG (panels in the upper row) and their corresponding DIC pictures (panels in the lower row) were obtained under various conditions as labeled on the top from flattened cochlear preparations. 2-NBDG was applied by cardiovascular perfusion. An arrow in A) points to the heads of pillar cells. Approximate borders of the cochlear regions are labeled by numbers next to the DIC image of A): inner hair cells (1), outer hair cells (2), Claudius cells (3), outer sulcus cells (4), spindle shaped cells (5), stria vascularis (6). E) Quantification of the relative 2-NBDG fluorescent intensity (Y-axis, normalized to the WT controls) in the Claudius cells (dark bars) and outer sulcus cells (empty bars) under various conditions (groups are labeled along the X-axis).

To directly test whether GJs facilitated glucose uptake from the blood circulation into the cochlear supporting cells, we perfused the 2-NBDG through the cardiovascular system. Uptake of 2-NBDG after systemic application was examined by measuring the intensity of 2-NBDG fluorescence in the supporting cells. In the WT mice, 2-NBDG fluorescence was clearly observed in the cochlear pillar cells (arrow in Fig. 6A), Claudius cells (region labeled 3 in Fig. 6A), outer sulcus cells (region labeled 4) and in the region of the stria vascularis (labeled 6 in the Fig. 6A, lower panel). NBDG fluorescence was relatively weak in the inner (region labeled 1 in Fig. 6A) and outer (region labeled 2 in Fig. 6A) hair cell regions. The specificity of 2-NBDG fluorescent signal was confirmed by using excess non-fluorescent glucose to compete with the 2-NBDG. A large molar ratio of glucose to 2-NBDG efficiently diminished the 2-NBDG fluorescence (Fig. 6C, upper panel). The loading of 2-NBDG was dramatically reduced in the cochlea of Cx30 null mice (Fig. 6B) or when the GJs were blocked by octanol (2 mM, Fig. 6D), supporting the hypothesis that the 2-NBDG uptake was dependent on the normal function of GJs. Quantification of the results showed that the 2-NBDG loading was reduced to 41.5±3.8% and 54.3±3.8% (n = 5) in the Claudius and outer sulcus cells, respectively, of WT control mice after GJs were blocked by 2 mM octanol (Fig. 6E). In the cochlea of Cx30 null mice where homomeric Cx26 GJs were still present (Fig. 2B), averaged 2-NBDG loading (n = 5) was 65.1±8.3% and 37.3±9.4% of the WT control values in the Claudius cells and outer sulcus cells, respectively. These values were further reduced to 6.3±4.9% and 3.6±5.2% (n = 5) when excess glucose was used to compete with the 2-NBDG in the systemic applications. In summary, our results indicate that GJs are required for efficient transfer of glucose from the blood circulation into the cochlear supporting cells, and the Cx30 null mutation significantly decreases this important GJ-mediated metabolic coupling in sensory epithelium of the cochlea.

**Figure 7:**
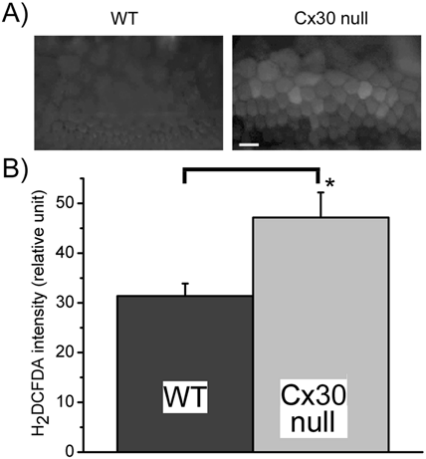
Comparison of intracellular ROS levels in the cochlear supporting cells of WT and Cx30 null mice. (A) A pair of images comparing H_2_DCFDA fluorescence obtained from supporting cells of WT (left panel) and Cx30 null (right panel) mice. (B) Quantification of the averaged intracellular H_2_DCFDA fluorescent intensity measured from WT and Cx30 null mice. The asterisk indicates a statistically-significant increase in the intracellular ROS level in the supporting cells of the Cx30 null mice.

Intracellular glucose deficiency is known to affect mitochondria function and increase the generation of reactive oxygen species (ROS) [23]. Therefore, we compared the level of ROS in the cochlear supporting cells of WT and Cx30 null mice (Fig. 7A). After ROS generation related intracellular fluorescent intensity was stabilized, we compared the H_2_DCFDA fluorescence from randomly-selected Claudius and outer sulcus cells (n = 278, from 6 mice). Comparing to WT mice, average intensity of CM-H_2_DCFDA fluorescence in these cells was 50.3% higher in the Cx30 null mice (Fig. 7B), which is statistically highly significant (p<0.01).

## Discussion

It has been more than ten years since genetic studies linked mutations in Cx26 [3], [4] and Cx30 [6] to human non-syndromic congenital deafness. Cx mutations are now widely accepted as one of the most common human mutations causing severe sensorineural deafness. Previous investigations using either conditional Cx26 knockout [7] or Cx30 null [8] mice revealed that the gross cochlear morphology was not changed by the deletion of either Cx gene. Major functional defects found by these studies are a reduced endolymphatic potential (EP) and apoptotic hair cell loss after the onset of hearing. The cellular mechanisms of Cx-mutation-linked deafness, however, are still unknown. One relatively new hypothesis is that the damaged microvessels in the stria vascularis provide a short-circuit leak conductance to reduce the endolymphatic potential [10]. A leading theory proposes that GJs in the cochlea provide intercellular ionic pathways for rapid removal of K^+^ ions away from the base of hair cells. Despite its popularity, the K^+^ recycling theory is only supported indirectly by experimental data [24], [25].

This study used Cx30 null mice to directly investigate the effects of deleting one of the two major cochlear Cxs on ionic and metabolic intercellular coupling in the cochlea. We focused on determining the effect of Cx30 null expression on the intercellular coupling among the cochlear supporting cells, which reside in the avascular sensory epithelium of the organ of Corti. Intercellular coupling provided by GJs in this region is suspected to facilitate transfer of nutritive molecules [18]. Fluorescent dye diffusion assay was used previously to study biochemical coupling in the cochlea [18], [26]. However, the morphological integrity for cochlear supporting cells is especially hard to preserve in all previous preparations. For this reason, GJ-mediated intercellular coupling among cochlear supporting cell in the sensory epithelium has not been systematically studied due to technical difficulties so far.

By using acutely prepared flattened cochlear samples in which all types of cells in the sensory epithelium, the lateral wall and stria vascularis were directly accessible to the microelectrodes (Fig. 1, Fig. 2C), we report for the first time that cochlear GJs allowed extensive intercellular biochemical coupling among the supporting cells in the organ of Corti. Both positively- (Fig.5, A&B) and negatively-charged (Fig. 5, C&D) molecules passed the GJs of cochlear supporting cells efficiently. 2-NBDG, a glucose analogue, diffused to >100 cells in a time course of about 20 minutes when it was injected into a single outer sulcus cell (Fig. 5F). The molecular weight of 2-NBDG (342.3) is larger than that of glucose (180.1). These data support that glucose is usually transferred intercellularly across many layers of cells in the organ of Corti. Our results further revealed significant differences in biochemical coupling between WT and Cx30 null mice, while the reduction in electrical coupling as a result of *Gjb6* gene deletion was statistically insignificant (Fig. 2). One possible explanation for the small change in whole cell G_j_ could be that the single channel conductance of Cx26 homomeric GJs was significantly larger than that of hybrid GJs consisting of Cx26 and Cx30. The increased single channel G_j_ could theoretically compensate for the apparent loss in the number of GJs as suggested by immunolabeling from the Cx30 null mice (Fig. 2, A&B), although this hypothesis will need to be tested experimentally.

Results obtained by systemic cardiovascular perfusion of 2-NBDG demonstrated that the uptake of glucose in the sensory epithelium of the cochlea is GJ dependent (Fig. 6). These data extended recent findings showing that GJs facilitate NBDG uptake in the lateral wall [27] and spiral limbus [28] of the cochlea. Combining these findings with our data directly showing that intercellular GJ-mediated 2-NBDG diffusion among supporting cells in the Cx30 null mutant mice was dramatically reduced (Figs. 4B, 5E & 5F) and intracellular ROS level was significantly increased (Fig. 7), these results suggest that cellular energy supply and metabolic homeostasis of the mutant cochlea could be affected in the regions that normally lack direct microcirculation. Therefore, our findings identified one important pathogenic process in the cochlea of the mutant mice. We believe these new findings laid a foundation for a new conceptual framework based on which Cx-mutation linked hearing loss may be explained.

### Experimental data do not support a role for disrupted intercellular ionic coupling as a major mechanism for cochlear dysfunctions caused by Cx mutations

The extracellular endolymphatic space in the cochlea is filled with an unusual fluid that is high in K^+^ (∼160 mM) and electric potential (∼+80 mV). Such an unusual ionic environment on the apical side of hair cells is essential for normal hearing, as it provides an energetically efficient and sensitive mechanism to transduce mechanical deflection of stereocilia into the receptor potential of hair cells [25]. Apical influx of K^+^ into hair cells is balanced by basal efflux. Although K^+^ recycling in the cochlea is a well-accepted idea [25], [29], [30], the route through which excess extracellular K^+^ around the base of hair cells is recycled back to endolymph is still under debate. Some investigators propose that the extracellular K^+^ is absorbed by supporting cells in the immediate vicinity of hair cells, then K^+^ is delivered through root cells and the type II fibrocytes via the epithelial cell GJ system back to the lateral wall [31], [32]. However, direct measurements of current flux support the concept that K^+^ enters the perilymph, flows toward spiral ligament in the scala tympani, and then is absorbed back into the connective tissue GJ system in the lateral wall [33]. These experimental data show that a significant portion of the K^+^ recycling bypasses the epithelial GJ system of the supporting cells in the sensory epithelium, which diminish the functional importance of the epithelial GJ system in the K^+^ recycling. However, targeted deletion of Cx26 specifically in the epithelial GJ system clearly shows that GJ coupling in cochlear supporting cells is required for normal hearing [7].

Recent data also challenged the theory that loss of K^+^ recycling underlies Cx mutation linked deafness. Immunolabeling of the cochlea of conditional Cx26 and Cx30 null mice showed that homomeric GJ plaques are extensively expressed in the cochlea [7], [8]. Boosting the protein level of homomeric Cx26 GJs is sufficient to rescue hearing of Cx30 null mutant mice [21]. Linkage of Cx26 mutations specifically affecting biochemical coupling (e.g., V84L, V95M, and A88S) to human deafness [15], [16] support the notion that disruption of GJ-mediated biochemical coupling alone is sufficient to cause deafness. Specific gating of GJ-mediated biochemical coupling has also been proposed recently [34]. Single channel recording data suggest that normal gating of GJs has little effect in regulating electrical coupling due to GJ channels dwelling in subconductance rather than closed state [35].

The developmental time point when K^+^ recycle becomes functionally required is not clear. However, it can be argued that considerable K^+^ recycling activity is not initiated until the endolymphatic potential is established at around P10 in mice [25]. In contrast, the efficient supply of glucose is always required. Mutational effects on biochemical coupling may therefore cause a chronic shortage in glucose supply that accumulates in early period of cochlear development before the K^+^ recycling becomes functionally required. The notion that Cx30 null mutation results in a disrupt of K^+^ recycling is not supported by our data (Fig. 2H) showing that the reduction in whole cell G_j_ between supporting cells in Cx30 null mice was statistically insignificant. In summary, although impairment in intercellular glucose transport through GJs does not necessarily rule out the K^+^ recycling theory entirely, available data and the new data presented here seem to favor that effects of Cx30 null mutation on biochemical coupling are the major cause for hearing loss observed in these mutant mice.

### Deficit in GJ-mediated metabolite transfer may be one important pathogenic process for Cx deficiency-linked deafness

In this study, we have for the first time demonstrated experimental evidence directly showing a dramatic reduction in GJ-mediated metabolic coupling among the cochlear supporting cells of Cx30 null mice. Since deficiency in GJ-mediated glucose transfer is known to underlie molecular mechanisms for functional defects including embryonic lethality in the Cx mutant mice [36], it is conceivable that a chronic glucose shortage exists in the cochlea of Cx30 null mutant mice. Lacking a direct microcirculation in the sensory epithelium would make the organ of Corti the most vulnerable region in the cochlea. Although most of the O_2_ consumed by mitochondria is reduced fully to water, ∼2% of electrons leak out in the oxidative phosphorylation chain and generate superoxide anions (O_2_^−^) and H_2_O_2_ during the electron flow. Decrease in glucose supply is a well-known cause exacerbating ATP exhaustion and increase of reactive oxygen species (ROS) [23]. Higher concentrations of ROS further impairs mitochondrial functions by damaging mitochondrial membranes resulting in the release of cytochrome C and the activation of apoptotic pathways, leading ultimately to cell death.

Based on the recently available data [7], [8], [13], [14], [15], [16], [27], [28] and results presented in this paper, we propose the following general scheme as a new theory to explain deafness caused by Cx mutations. Loss of function Cx mutations cause sufficient decrease in GJ-mediated metabolic coupling in cochlear regions where microcirculation is poor and sufficient supply of glucose normally depends on GJ-mediated intercellular transfer. Reduction in glycolysis decreases availability of ATP, which increases the mitochondrial ROS production. The broad spectrum of damaging effects caused by increased intracellular ROS ultimately leads to functional failure and cell death in the sensory epithelium of the cochlea. Experiments are now being undertaken in our lab to test many aspects of this novel hypothesis regarding the mechanisms for deafness in the conditional Cx26 knockout and Cx30 null mice.

## Materials and Methods

### Flattened cochlear preparations

Immunolabeling data demonstrated that GJs in the cochlear supporting cells have matured to an adult-like pattern [20] around postnatal day 8 (P8). Therefore, we used littermate-controlled WT and Cx30 null [8] mice between the age of P8–P12 in this study. The genotype of WT and Cx30 null mice was determined by PCR genotyping as previously described [8]. The animal use protocol was approved by the Emory Institutional Animal Care and Use Committee. Mice were anesthetized using xylazine (i.p., 10 mg/kg) and ketamine (i.p., 100 mg/kg) before they were killed by decapitation. Both temporal bones were rapidly removed and immersed in ice-cold Hanks' balanced salt solution (HBSS, Sigma Aldrich, MO). Observed under a dissection microscope (Stemi DV4, Zeiss, Germany), the bony capsule of the cochlea was removed with the tip of a 28G needle. Starting from the apical turn, we removed the Reissner's membrane from all the cochlear turns with a pair of fine forceps. The lateral wall was kept intact during microdissection. The dissected cochlear turns was cut into ∼2 mm segments. The cochlear segments were flattened by placing two micro glass rods at the two ends of the segment. The glass rods were made from broken tips of regular patch-clamp microelectrodes, which were mounted on a drop of sylgard (Dow Corning Corporation, KY) adhered to a glass cover slip. The angle of the mounting made the micro glass rods behave like spring hinges with a downward pressing tendency. Fig. 1A gives an illustration of a fresh flattened cochlear preparation when viewed with differential interference contrast (DIC) optics. Based on characteristic cell shape, size and their relative locations, cochlear cells in the sensory epithelial, lateral wall and stria vascularis are clearly identifiable in this live tissue preparation (various cell types are marked in Fig. 1,A&B). A fixed preparation immunolabeled with FITC-conjugated phalloidin to outline the border of various types of cochlear cells is given in Fig. 1B. This procedure was used in some experiments to help us identify and count the cells transferred fluorescent dyes when they were injected into a single cell (Fig. 3, A, E&G).

### Immunolabeling methods applied to the flattened cochlear preparation

Flattened cochlear preparations were fixed in 4% paraformaldehyde (in phosphate-buffered solution, PBS) for 30 minutes. Samples were permeablized in 0.1% triton (in PBS) for 30 minutes and blocked in 10% goat serum (in PBS) for 1 hour. Depending on the purpose of the experiment, following primary antibodies were used: (1) Cx26 (catalog# 71-0500, lot#370925A, dilution 1∶200, 4°C overnight, Zymed Laboratories, CA); (2) Cx30 (catalog# 71-2200, lot#354025A, dilution 1∶100, 4°C overnight, Zymed Laboratories, CA). After washing with PBS three times, samples were incubated with FITC-conjugated (catalog# 111-096-003) goat anti rabbit secondary antibodies (Jackson ImmunoResearch Lab, West Grove, PA) for 2 hours (at room temperature) to visualize immunolabeling results. Some of the dye-injected samples were double labeled with either FITC- or Cy3-conjugated phalloidin (catalog#P-1951, lot#093K0474, dilution 1∶1000, Sigma, MO) for 30 minutes to delineate the cell border (Figs. 1&2). All samples were mounted in fluoromount-G antifading solution (catalog#17984-25, Electron Microscopy Science, PA) and examined with a confocal microscope (Zeiss LSM, Carl Zeiss, Germany). Layers of optical sections were superimposed to show a more complete pattern of immunolabeling through multiple layers of cells in the flattened cochlear preparation.

### Double electrode patch clamp recording performed with the flattened cochlear preparation

A detailed protocol for double electrode patch clamp was described previously [15]. Briefly, each cell in the cell pair was voltage clamped independently with an Axopatch 200B amplifier (Axon Instruments Inc., Foster City, CA) by following a conventional whole cell voltage clamp method. Signals amplified by Axopatch 200B amplifiers were low-pass filtered at 1 kHz and digitized at 5 kHz. Experiments were controlled by Clampex software (version9.2) via a Digidata 1322A interface (Axon Instruments Inc., Foster City, CA). Holding voltage (V_h_) is −70 mV for both cells. The voltage in one of the cells (V_1_) was stepped between −110 mV and −30 mV in a step of 10 mV for one second, while the membrane potential of second cell in the pair (V_2_) was held at −70 mV. A transjunctional voltage (V_j_ = V_1_−V_2_) is therefore established, which resulted in junctional currents (I_j_) that were measured in the unstepped cell. The transjunctional I–V curve was obtained by plotting the peak I_j_ (y-axis) against the V_j_ (x-axis). The junctional conductance (G_j_) was calculated by: G_j_ =  I_j_/V_j_. The GJ uncouplers, octanol and FFA were both purchased from Sigma Aldrich Inc. (St Louis, MO). Data were analyzed using pClamp (version 9.2) and Origin 7.0 (OriginLab, Northampton, MA). The data points were presented as means±standard error (S.E). All data were statistically compared by student *t* tests and a significance level of p<0.05 was used.

### Fluorescent dye diffusion assay performed with the flattened cochlear preparation

Flattened cochlear preparations were placed in a recording chamber mounted on an upright microscope (Axioskop2 FX plus, Carl Zeiss, Germany). Extracellular solution (HBSS) was perfused at a rate of ∼1 drop/second. DIC optics enabled us to directly recognize different cochlear cell types in the acutely-isolated cochlear segment (Fig. 1A). Membrane impermeable fluorescent dyes were injected into the cytoplasm of a single cell by forming the whole-cell patch-clamp recording mode, which was confirmed by monitoring the whole cell resistance and capacitance using the standard protocol (Axonpatch 200B, Axon Instruments, CA). Pipettes had an access resistance of about 3 MΩ. Analog signals were digitized with the Digidata 1322A interface (Axon Instrument, CA).

Pipette solution contained (in mM): 120 KCl, 1 MgCl_2_, 10 HEPES, 10 EGTA, pH adjusted to 7.2 with KOH. Fluorescent dye (all purchased from Molecular Probes, OR) supplemented in the pipette solution was one of the following: (1) 1 mM lucifer yellow (LY, lithium salt, MW = 457 Da, charge = −2, catalog#L-453); (2) 0.75 mM propidium iodide (PI, MW = 668 Da, charge = +2, catalog# P1304MP); (3) 1 mg/ml AlexaFluor 568 (hydrazide sodium salt solution, MW = 791.8 Da, charge = −1, catalog#A-10441); (4) 200 µM 2-(*N*-(7-nitrobenz-2-oxa-1,3-diazol-4-yl)amino)-2-deoxyglucose (2-NBDG, MW = 342.3 Da, catalog# N13195). Intercellular dye diffusion patterns were recorded by a cooled digital CCD camera (AxioCam, Carl Zeiss, Germany) at various time points after the establishment of whole cell recording configuration. The image acquisition was controlled by AxioVision 3.1 software (Carl Zeiss, Germany) and the same exposure time was used for samples obtained from WT and Cx30 null mice. Dye recipient cells were visually identified (Figs. 3&4) using the same criteria in the images of WT and Cx30 null mice. Either the nuclei or the cell border must be clearly discernible, and their numbers were counted from these images. All experiments were carried out at room temperature (20–25°C).

### Assessment of in vivo 2-NBDG uptake in the supporting cells after systemic applications

Mice were anesthetized by injections of xylazine (i.p., 10 mg/kg) and ketamine (i.p., 100 mg/kg). A shielded winged needle (Becton Dickinson Infusion Therapy System Inc., Franklin Lakes, NJ) was penetrated into the left ventricle to introduce solutions into the cardiovascular system of mice. The speed of the perfusion was controlled by using a peristaltic pump (Instech Laboratories Inc, Plymouth Meeting, PA). Animals were divided into four experimental groups: (1) WT mice in which 3.8 mM of 2-NBDG (in HBSS) was perfused for 10 minutes; (2) WT mice in which 2 mM of octanol (in HBSS, catalog#: O-4500, Sigma-Aldrich, St Louis) was perfused first for 10 minutes, then followed by another 10 minutes of 3.8 mM of 2-NBDG plus 2 mM of octanol (in PBS) perfusion. To exclude the possibility that the change in the 2-NBDG distribution in octanol-treated mice was due to the cardiac output decrease, we performed the heart massage during the injection of 2-NBDG until the end of the experiment to maintain systemic circulation. The presence of 2-NBDG fluorescence (Fig. 6D) in stria vascularis confirmed that the fluorescent tracer reached the cochlea. (3) WT mice in which 3.8 mM of 2-NBDG plus 0.5 M of glucose (in PBS, catalog#: G-7528, Sigma-Aldrich Inc, St Louis) were perfused for 10 minutes; (4) Cx30 null mice in which 3.8 mM of 2-NBDG (in HBSS) was perfused. Concentrations of NBDG used in these groups mimics the glucose concentration in the plasma and cochlea [37].

During dissection and preparation of the samples 2 mM octanol was always present in the solution to prevent the loaded 2-NBDG from leaking out of the opened GJ hemichannels. Samples were then transferred to perfusion chamber mounted on stage of an inverted fluorescent microscope (Axioskop 2 FS plus, Carl Zeiss, Germany) for image acquisition. To minimize photobleach, we used a shutter wheel (LAMBDA 10-B, Sutter Instruments Inc, Novato, CA) in the light path to expose the samples to the intense fluorescent light source only during the image acquisition. To ensure fluorescent intensities are comparable across different samples, a constant exposure time (300 mSec) was used. Images were taken with a cooled-CCD camera (AxioCam MRm, Carl Zeiss, Germany) under the control of AxioVision software (version 4.6, Carl Zeiss Inc, Germany). Pictures were stored in the computer hard disk for offline analysis. The fluorescent intensity of the area of interest was measured with the ImageJ software (version 1.40 g) we downloaded from http://rsbweb.nih.gov/ij/.

### Assessment of intracellular ROS level

The level of intracellular ROS was monitored with a fluorescent indicator dye CM-H_2_DCFDA (Molecular Probes, CA) [38], [39]. CM-H_2_DCFDA is the acetoxymethyl ester form of H_2_DCFDA, which is cell-permeant and non-fluorescent until removal of the acetate group by ubiquitous intracellular esterase and oxidation occurs within the living cells [38]. The molecule then becomes highly fluorescent 2′,7′-dichlorodihydrofluorescein diacetate. The absorption and emission properties of fluorescent H_2_DCFDA are similar to FITC. Our time-lapsed recordings showed that the intracellular fluorescent intensity of H_2_DCFDA in the cochlear supporting cells was stabilized when these cells were incubated in H_2_DCFDA (10 µM) for about 70 minutes, suggesting that the intracellular dye loading was completed in such a time period. After 80 minutes incubation in 10 µm of H_2_DCFDA, images were taken with an AxioCam digital camera (AxioCam MRm, Zeiss, Germany) controlled by software AxioVision 4.6 (Zeiss, Germany). Same exposure time (20 mSec) was used for image acquisitions of WT and Cx30 null mice. Photobleaching was minimized by exposing the cells to the excitation light only when images were acquired by controlling an electronic shutter (Lambda 10, Sutter Instrument, CA) placed in the light path. ROS generation-correlated fluorescent intensities in the supporting cells were measured from individual cells by the ImageJ software and averaged results were presented. Data obtained from WT and Cx30 null mice were compared by student *t* tests with a significant level set at p<0.05.

## Supporting Information

Figure S1 Co-immunolabeling of Cx26 and Cx30 in the cochlea of Cx30 null mice. Images were obtained with a conventional fluorescent microscope at the same focus plane by four different optical settings from the same sample. (A) Immunoreactivity of Cx26 showing the remaining GJ plaques in the Claudius cells (one example is given by a small white arrow) and outer sulcus cells (white arrowhead) in the cochlea of the Cx30 null mice. (B) Immunoreactivity of Cx30 obtained from the same view as that in (A). Focus was not changed. The antibody against Cx30 was confirmed to generate positive results in the WT animals. (C) Differential interference contrast (DIC) image of the same cochlear tissue as that obtained in (A). Focus was not changed. Regions of the outer sulcus cells and Claudius cells are labeled. (D) Fluorescent image of DAPI showing the nuclei of the cochlear cells. The image was obtained from the same cochlear tissue as that in (A) with the same focus. Borders of the region of outer sulcus cells and Claudius cells are outlined by white lines and labeled.
(4.69 MB TIF)
