# Supplementary figures and images for: Gap Junction Mediated Intercellular Metabolite Transfer in the Cochlea Is Compromised in Connexin30 Null Mice
Source: PLoS One. 2008 Dec 31;3(12):e4088. doi: 10.1371/journal.pone.0004088 (PMC2605248; doi:10.1371/journal.pone.0004088)

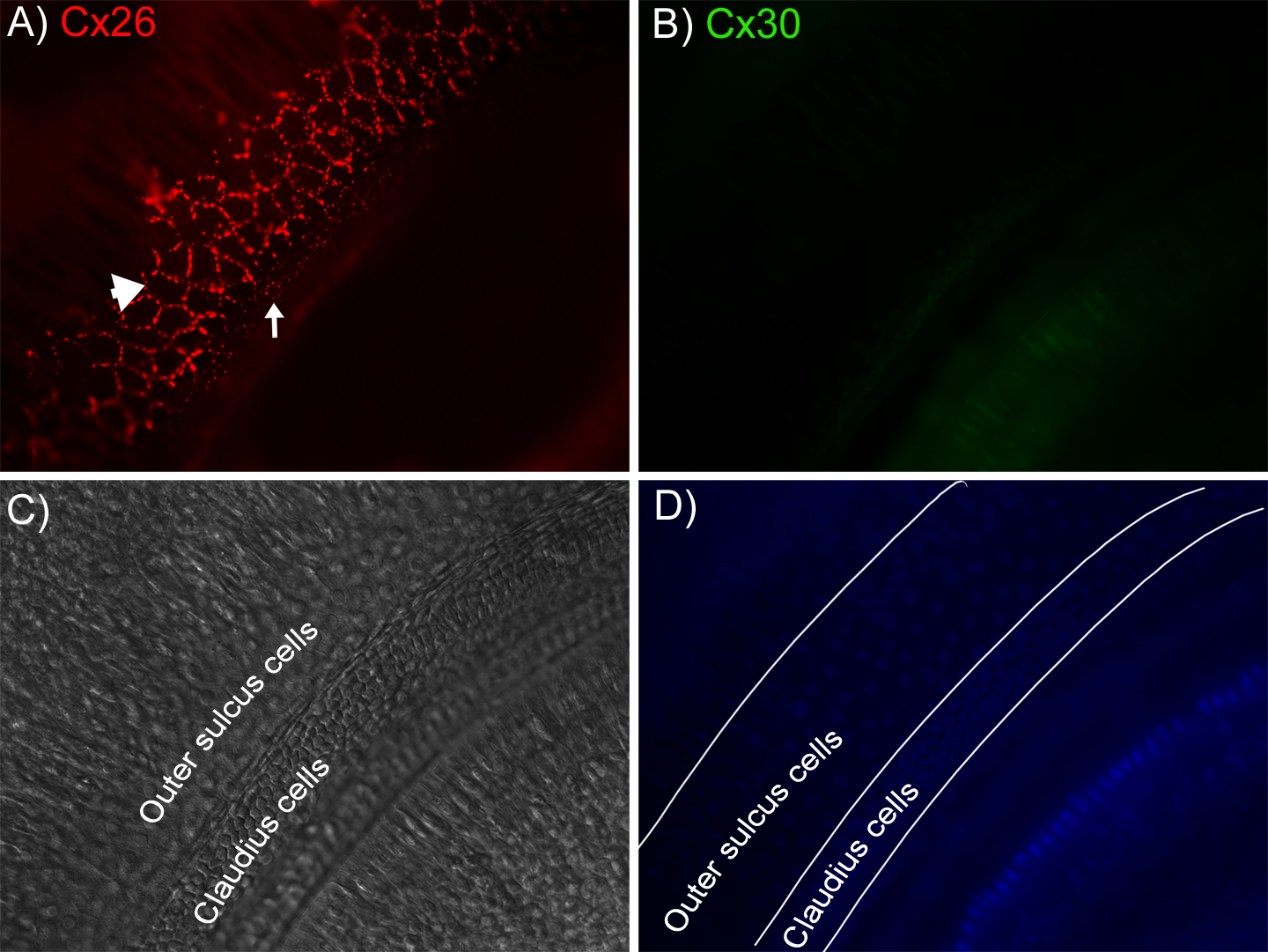

Supplement: Figure S1 — Co-immunolabeling of Cx26 and Cx30 in the cochlea of Cx30 null mice. Images were obtained with a conventional fluorescent microscope at the same focus plane by four different optical settings from the same sample. (A) Immunoreactivity of Cx26 showing the remaining GJ plaques in the Claudius cells (one example is given by a small white arrow) and outer sulcus cells (white arrowhead) in the cochlea of the Cx30 null mice. (B) Immunoreactivity of Cx30 obtained from the same view as that in (A). Focus was not changed. The antibody against Cx30 was confirmed to generate positive results in the WT animals. (C) Differential interference contrast (DIC) image of the same cochlear tissue as that obtained in (A). Focus was not changed. Regions of the outer sulcus cells and Claudius cells are labeled. (D) Fluorescent image of DAPI showing the nuclei of the cochlear cells. The image was obtained from the same cochlear tissue as that in (A) with the same focus. Borders of the region of outer sulcus cells and Claudius cells are outlined by white lines and labeled. (4.69 MB TIF) [file pone.0004088.s001.tif]
